# Verbal Information From Parents About Stillbirth: The VIPS Study (Phase One)

**DOI:** 10.1111/ajo.70018

**Published:** 2025-04-11

**Authors:** Azriel Gan Lin Lo, Lauren J. Breen, Zoe Bradfield, Scott White, Sonya Criddle, Georgia Griffin, Bligh Berry, Jane Warland

**Affiliations:** ^1^ School of Population Health and Curtin enAble Institute Curtin University Perth Western Australia Australia; ^2^ School of Nursing Curtin University Perth Western Australia Australia; ^3^ King Edward Memorial Hospital Perth Western Australia Australia; ^4^ Division of Obstetrics and Gynaecology, UWA Medical School The University of Western Australia Perth Western Australia Australia; ^5^ PathWest Perth Western Australia Australia; ^6^ Adelaide University Adelaide Australia

**Keywords:** bereaved parents, cause of death, Delphi, Intrauterine Fetal Death, stillbirth

## Abstract

**Background:**

In many countries, a baby's cause of death (COD) following stillbirth is informed only by case notes and pathology investigations. However, parents' understanding of their baby's COD may inform or even change the COD diagnosis. We aimed to produce a standardised co‐designed interview schedule to enable parents to contribute information to improve overall understanding of the causes of stillbirth.

**Materials and Methods:**

Consensus for the interview schedule was sought via a two‐round modified Delphi study. We recruited internationally for panel membership comprising bereaved parents, clinicians, and researchers. In Round 1, each panellist provided up to five questions to ask bereaved parents. After collation into categories, Round 2 asked panellists to rate the importance of each question category on a four‐point scale.

**Results:**

Panellists (*n* = 126 Round 1, *n* = 75 Round 2) were mainly bereaved parents. In Round 1, 553 potential interview questions were generated. These were grouped into categories which were pregnancy experience, antenatal care, fetal wellbeing, maternal wellbeing, days Prior to stillbirth and perceived COD. These proposed questions and question categories were then put to panel members in Round 2. All categories achieved consensus for inclusion in the final interview schedule with positive consensus percentage scores ranging from 83% to 98%.

**Conclusions:**

Panel membership comprising mainly bereaved parents provided a clear mandate for questions parents want to be asked. The interview schedule will soon be trialled with recently bereaved parents at a tertiary‐referral maternity health service. Findings from the study will inform future research on how to include parents' voices in COD determination.

## 1 Introduction

The definition of stillbirth varies across the globe, for example in Australia it is said to occur when a baby is born without showing signs of life from 20 weeks gestation or weighing 400 g or more [1] whereas in the UK the gestational age is 24 weeks. In many parts of the world when assigning cause of death (COD) for stillbirth, it is usual for a hospital‐based perinatal review panel to meet and assign COD based on local perinatal COD guidelines (e.g. ICD‐10) [2]. This meeting does not usually include a parent interview [3]. Indeed, a study involving six high‐income countries reported that it is common for parents to be unaware that a perinatal review of their baby's death had even occurred [4]. This means that other than providing consent for investigations, such as autopsy and blood tests, parents are often not given an opportunity to provide any other information about events that may have occurred during the pregnancy or in the days and weeks leading up to their baby's death. However, information such as this may provide important insights into how and why the baby died.

Findings from Bakhbakhi et al. ‘PARENTS’ studies [5, 6, 7, 8] in the United Kingdom provided significant support for the basis of our study. The focus of these studies was to explore “whether and how” parents could be involved in perinatal mortality review, to provide them with an avenue to give feedback to the health service about their care and ways to identify and address sub‐standard care. Their work has provided assurance for the value of parents' participation in perinatal mortality review; the authors concluded that parental engagement in perinatal review may provide information not captured in case notes that may assist in COD determination or identify care and system delivery problems for further improvement. However, there remains significant lack of understanding of the role that a parent interview could play in shedding further light on COD determination post‐stillbirth.

Providing the opportunity for parents to tell their story while also collecting information to provide insight into what caused the baby's death, and contribute to the body of knowledge about novel risk factors for stillbirth, are important reasons why undertaking a parent interview following stillbirth may be invaluable. It is time to give a voice to parents, to listen to them and value their knowledge and expertise following stillbirths, while providing an evidence‐based, co‐designed, rigorously tested interview schedule to collect this information. By taking a methodologically rigorous consensus driven approach [9], we aimed to create an interview schedule to collect parents' perspectives on their baby's COD.

## 2 Materials and Methods

### 2.1 Design

This paper outlines the first phase of a two‐phase study, in which a modified Delphi‐type approach [10] was used to gain consensus from international experts on questions that should be included in an interview schedule following stillbirth. The Delphi methodology is a structured multistage process, providing a rigorous way of determining consensus on any given topic from a diverse group of experts [10]. The approach is an efficient, effective way to engage with a large group of experts because it overcomes potential issues related to large group dynamics such as “groupthink” [11]. In this study, we utilised anonymous online surveys to capture expert panellist opinions individually and anonymously.

We utilised two survey rounds, both rounds were delivered using Qualtrics^XM^ software [12]. In the first round, panellists were asked to provide up to five questions that parents should be asked in an interview post‐stillbirth to explore their baby's COD. Following the first round, we merged similarly themed questions into question categories and then, we asked panellists to provide a rating of importance for each question category. This was to gather consensus from panellists on the importance of including each in the interview schedule.

### 2.2 Ethical Considerations

Institutional ethics approval was granted by Curtin University Human Research Ethics Committee (HRE2022‐0392). Participants provided consent via a hurdle question, indicating that they fulfilled the inclusion criteria, had read the participant information sheet linked to the survey, and agreed to participate. In each round, participants were asked for demographic information and then to complete the survey. At the beginning and end of the survey, participants were provided with a list of support agencies and resources about stillbirth should they need them. They were also invited to provide their email, via a separate survey link, if they wished to participate in later rounds.

### 2.3 Panellists

Eligible panellists were 18 years or above, and self‐identified as belonging to one of the following panels—parents bereaved by stillbirth, clinicians providing maternity care, and/or researchers/policymakers whose works were related to stillbirth. All panellists were asked (1) which panel they wished to join and (2) their country of residence (see Table 1).

**TABLE 1:** Rounds 1 and 2 participant demographics.

Round 1 panellist country of residence and panellist type			
Country of residence	Bereaved parents *n* = 109	Maternity clinicians *n* = 11	Researchers and policymakers *n* = 6
USA	103	6	4
Canada	4	0	0
Australia	2	1	1
Not specified	0	4	1

Round 2 panellist country of residence and panellist type^a^			
Country of Residence	Bereaved Parents *n* = 65	Maternity clinicians *n* = 6	Researchers and Policymakers *n* = 3
USA	59	2	3
Canada	3	0	0
Australia	3	1	0
Not Specified	0	3	0

^a^ In Round 2 one panellist did not indicate their panellist type.

Panellists were recruited through convenience sampling [13]. The study was advertised through social media (e.g. Twitter, Face‐book and Instagram), word‐of‐mouth and targeted email recruitment (such as the Australian College of Midwives). Panellists from Round 1 were invited to participate in Round 2. Different from the traditional Delphi study approach, participants were not necessarily retained for both rounds. Potential panellists may not have felt able to provide a potential question in Round 1, but could contribute to consensus in Round 2. Due to participant anonymity, we do not know how many panellists took part in both rounds. Recruitment for Round 1 occurred from 1 August to 30 August 2022, and Round 2 occurred from 1 November to 30 November 2022.

### 2.4 Delphi Rounds and Analysis

In Round 1, participants were asked, *“*If you could ask five questions of a bereaved parent about their experiences leading up to their baby's death, to help determine the cause of death, what would those questions be?*”*. Participants were provided with open text boxes to respond.

**FIGURE 1:**
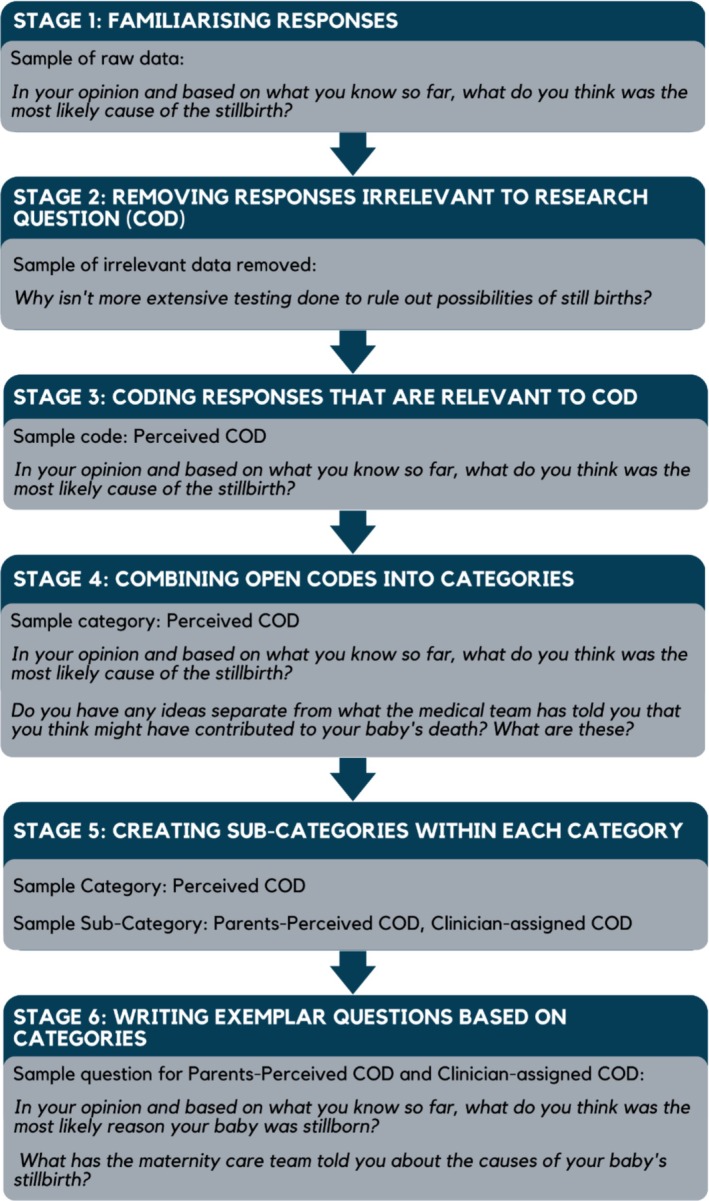
Round 1 analysis: modified inductive content analysis steps. The figure outlines the analysis steps and exemplar raw data, categories, sub‐categories and questions included in the interview schedule. Content Analysis steps were based on Kyngäs, H. (2020). Inductive Content Analysis. In H. Kyngäs, K. Mikkonen, & M. Kääriäinen (Eds.), *The Application of Content Analysis in Nursing Science Research* (pp. 13–21). Springer International Publishing. https://doi.org/10.1007/978‐3‐030‐30199‐6_2.

Over 550 suggested questions were received. Many of these were similar. The first and last author met to collate and condense question categories and subcategories for each section of the interview schedule and to decide on which question or questions might be best to use in the interview schedule. To achieve this, they took a modified inductive content analysis approach [14] as shown in Figure 1. Once determined, these questions and question categories were then shared with the rest of the team to gain their agreement.

Using this process, the initial 553 questions were collated and condensed into six question categories:

1. Pregnancy Experience (the everyday experience of pregnancy from the parents' point of view).
2. Antenatal Care (perception of pregnancy care provided by a maternity care provider).
3. Fetal Wellbeing (perceived or told understanding of fetal health and/or ill health).
4. Maternal Wellbeing (perceived or told understanding of pregnant person's health).
5. Days prior to Stillbirth (events which may have heralded the stillbirth), and
6. Perceived COD (parents' understanding of the cause of the stillbirth).

In Round 2, we presented the six categories with a description (see Table 2), along with some proposed questions. Unlike a traditional Delphi approach, individual questions were not rated separately. This was because we did not consider that asking our panellists to rate the importance of multiple, very similar, questions about the same thing would be useful for the development of an interview schedule. Instead, we asked our panellists to rate the importance of including each question category in the interview, on a Likert scale of 1–4 (*1 = not important to ask, 2 = good to ask; 3 = important to ask; 4 = vital to ask*). They were also provided with proposed questions and given an opportunity to change the wording or add any other questions they thought should be included in each category.

**TABLE 2:** Round 2 survey response summary.

Question‐category	Category descriptor	Proposed question/s from Round 1 put to panellists in Round 2	Panellist comments regarding proposed questions	Positive consensus Score %^a^
Pregnancy experience	Category includes questions to capture the everyday experience of pregnancy from the parents' point of view	*How would you characterise your pregnancy up until your baby's birth?* *What is something that stood out?*	*How was the beginning of your pregnancy?’ is better than asking how it was in general. We carried our babies for months, loved them, knew them, got excited, threw baby showers, bought baby items. Then they died. If someone asked how my pregnancy was it would be hard to feel any other way besides ‘tragic’* *Maybe ask, ‘how was your pregnancy up to your loss?’ because the experience of pregnancy changes drastically when a loss is known*	83.1%
Antenatal care (or pregnancy care)	Category includes questions to explore the care they received when they attended a visit with their maternity care provider	*How would you characterise your care? How well do you feel you were heard? Did you feel any appointments stood out, and if so why?*	*This is a very vital question to ask, because when people hear my story about how I lost my son, I tell them how it started and they would say ‘oh but you had the best care I bet,’, when in fact I did not receive the best care. Don't be afraid to go more in depth with the follow up questions after this one*	95.8%
Fetal wellbeing	Category includes questions on baby's wellbeing throughout pregnancy, including whether parents have concerns about their baby's movement (strength, frequency, pattern, hiccups) and other aspects of baby's health (concerns about the cord, placenta, baby's position, growth, and size). It encompasses any tests and screens undertaken in the pregnancy	*Did you ever have any concerns about your unborn baby's health/well‐being?* *Did you notice a change in fetal movement whether a decrease or increase in your baby's normal pattern of movement?*	*I think this is the most important set of questions*. *I think it's important to be specific about what is meant by fetal wellbeing. If related to fetal movement, this is critical—and additional information should be asked including whether there were changes in movement (both reduced and frantic movement), as well as what the health care team's response was to this change in movement* *I would suggest careful consideration about how these kinds of questions are asked and the intended use of the data. Given the self‐blame after stillbirth, it would be important to be sensitive to how women might perceive these questions*	96.2%
Maternal wellbeing	Category includes questions on maternal wellbeing throughout pregnancy, including signs and symptoms they had, any infections they experienced, any life‐style related factors (e.g. sleep, diet, activity, alcohol and drugs), and any physical injuries during the pregnancy. We will also ask about their mental wellbeing, including if they had a gut feeling that something was wrong	*What symptoms did you have throughout the pregnancy? D*id you ever have a gut feeling that something was wrong?	*I think the ‘gut feeling’ is one of the most important aspects to listen to. I knew my son had passed before it was confirmed by any type of test. If caregivers listened to a woman's gut feeling I believe there would be more opportunities to save stillborn babies. Women's intuition should never be fobbed off in this situation*.	91.5%
Days prior to stillbirth	Category includes questions to explore changes the pregnant person experienced in the days (approximately 14‐day period) before the stillbirth, including any perceived changes in fetal wellbeing, any changes, and any approaches they took when these changes were noticed	*Did you experience any change in pregnancy symptoms in the days preceding your loss?*	*This is vital because [of] mothers like me. I had a complete placental abruption and the last 2 weeks there were a lot of changes*.	90.1%
Perceived COD	Category includes questions to determine what parents believed caused their baby's stillbirth as distinct from what they may have been told	*In your opinion and based on what you know so far, what do you think was the most likely reason your baby was stillborn?* *What has the maternity care team told you about the cause of your baby's stillbirth?* *How much do you agree/disagree with what you were told?* *Is there anything you think you (or someone else) might have done that could have changed the outcome for your baby?*	*Very, very important. Also, if the mother or father have any questions about the cause of death and evidence found, (in my case, my placenta was calcified and aged beyond 19 weeks, I never got results on the testing I wanted done on my placenta and my baby)*	91.7%

^a^ Positive consensus is the sum of percentages from Ratings 3 and 4.

No questions were added to the proposed schedule after Round 2. The interview schedule was then reviewed and finalised by all authors. Please note this schedule (see supplementary files) is provided by way of information. We do not recommend its use in its current form because it will be further refined following testing with parents in the next stage of this two‐phase project.

## 3 Results

### 3.1 Participant Demographics

In Round 1, 254 participants opened the survey. One response was removed as they ticked that they did not consent to participate. Another 127 responses were removed as they provided demographic information only (i.e. they did not provide any potential questions). A final number of 126 panellists provided potential questions (parents = 109; clinicians = 8; researchers and policymakers = 3; see Table 1).

In Round 2, 84 potential participants opened the survey. Seven responses were removed as they did not provide a rating for any category or question. Two responses were assumed to be from the same person, because they were identical, so we merged their responses into a single entry. A final number of 75 participants provided Round 2 information (parents = 65; clinicians = 6; researchers and policymakers = 3; did not indicate = 1; see Table 1).

### 3.2 Results From Delphi Rounds

A total of 553 questions were collected from Round 1. Six broad question categories were created namely: pregnancy experience, antenatal care, fetal wellbeing, maternal wellbeing, days prior to stillbirth, and perceived COD. Each of the question categories had sub‐questions within the category. The number of questions per category varied from 27 (Pregnancy Experiences) to 172 (Fetal Wellbeing) and some proposed questions included components of more than one category (e.g. combining fetal wellbeing and events occurring in the days prior to the birth). There were also some questions proposed that were not relevant, for example panellists proposed questions about experiences of grief.

**FIGURE 2:**
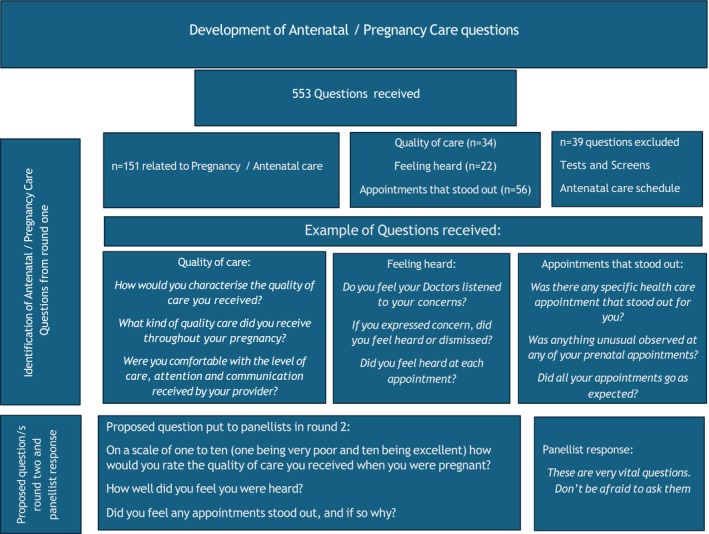
Development of antenatal/pregnancy care questions.

Figure 2 provides an example of how the questions in the antenatal care category in the interview schedule were developed from the 553 questions. We followed this same approach for each of the interview schedule question categories.

As the figure shows overall there were 151 questions that were related to Antenatal care. These fell into four sub‐categories:

- 34 related to quality of care.
- 22 related to feeling heard.
- 56 related to appointments that stood out.
- 39 related to specific tests and screens related to antenatal care schedule.

There were 34 questions the panellists suggested which related to quality of care. We considered that asking a Likert scale question followed by a request for an explanation of that rating would provide future interviewees with the opportunity to both characterise and also explain their perception of the quality of care they received. Therefore, the proposed question suggested in Round 2 was on a scale of one to ten (one being very poor and 10 being excellent) how would you rate the quality of care you received when you were pregnant? To gain more detail about this we added “please provide a rationale for this rating” to the final interview schedule.

The questions panellists supplied relating to feeling heard were all remarkably similar in their suggested wording (Figure 1) as well as being highly reflective of reports about being heard from bereaved parents experiences [15]. We therefore included the proposed question for panellists review in Round 2 as “How well did you feel you were heard?” and in the final interview schedule we added further clarity to this question by asking “Overall, how would you rate how well you felt you were heard by your maternity caregivers (doctors, nurses, midwives)?” (1 = not heard at all, 10 = all concerns heard and addressed) followed by a separate question: Would you like to tell me what prompted this rating?

Appointments that stood out was also a question that recurred in many of the proposed questions from Round 1. The questions panellists formulated ranged from a broad question such as “Was there any specific health care appointment that stood out for you?” to a somewhat more specific question such as “Did you have any concerns regarding the any visits you had from your healthcare providers? (anything missed, overlooked, potentially harmful, etc.)”. The team determined that a good way to ask for this information in the interview would be “Did you feel any appointments stood out, and if so why?” while this was endorsed in Round 2, to prevent a double barrelled question in the schedule the final interview questions were: During your antenatal visits were there any specific appointments that stood out for you? and, if Yes, can you tell me about one or two of these appointments?

We excluded the final sub‐category, which related to specific tests and screens as well as antenatal care provision, from Round 2. Examples of questions that were excluded were: how many appointments did you go to? Were you seen by a doctor or nurse? How many ultrasounds did you have? Did you have the glucose tolerance test? The reason for their exclusion was twofold, firstly these questions would have introduced unnecessary focus on country or centre specific variance in usual pregnancy management unrelated to the reason for the couple's stillbirth. Second, many tests and screens are offered routinely and are thus part of standard antenatal care [16]. So rather than asking a barrage of questions about optimal antenatal care we considered asking about appointments that stood out, as a better way of capturing anything that might have occurred during their visits that parents considered somehow unusual and therefore may be related to their baby's stillbirth.

Table 2 provides further detail on questions panellists were asked and their feedback for each question category.

To calculate the consensus for each question category, we combined the percentages of ratings of “3–important” and “4–vital to ask” assigned by the panellists for each category. This combined percentage represents the level of ‘positive’ consensus reached for each category. We considered positive consensus had been achieved if this combined rating was greater than 50%.

**FIGURE 3:**
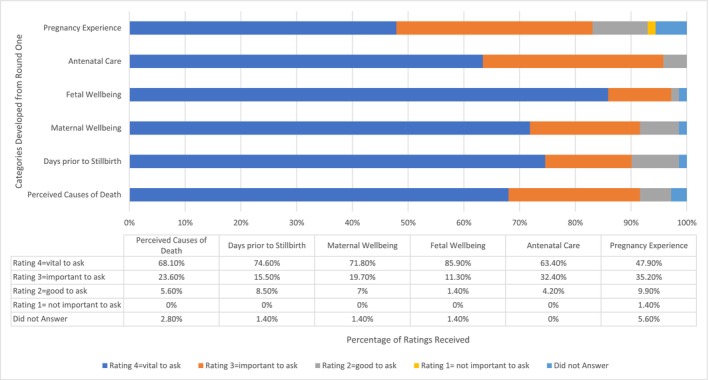
Round 2 results: percentage of ratings for each category. The developed question categories were presented to panellists in Round 2. This figure shows the percentage of each rating (1 = not important to ask, 2 = good to ask, 3 = important to ask, 4 = vital to ask) for each category respectively. The “consensus” is calculated based on the total percentage of Ratings 3 and 4 for each category.

The level of consensus for each question category in the interview schedule ranged from 83.1% (Pregnancy Experience) to 96.2% (Fetal Wellbeing; see Figure 3). Figure 3 provides a summary of results showing panellist rating for each category.

## 4 Discussion

This Delphi study had two rounds, from which we constructed an interview schedule to pilot in Phase 2 of this study. High participation in and consensus from parents bereaved by stillbirth in both rounds provides a strong mandate that the questions generated are those that parents want to be asked and, therefore, should be asked in determining COD following the stillbirth of their baby.

There is a paucity of research regarding common pregnancy experiences being included in current stillbirth investigation. Indeed, our panellists only proposed 27 potential questions in the *pregnancy experience* category in Round 1. As Figure 3 shows, this question category provided the lowest level of consensus for inclusion in the interview schedule. In order to keep the interview schedule to a manageable length and give preference to the most highly rated questions from Round 2, we therefore included just one short answer question in this category. Further, asking a short answer question about pregnancy experience at the beginning of the interview allows interviewers to build rapport with interviewees before the more detailed investigative questions are asked.

In contrast, antenatal care was ranked the second most important category with many potential questions supplied by panellists. A Cochrane systematic review [17] reports that optimal antenatal care and timely interventions have a significant impact on preventing stillbirth. However, in most reports, the quality of antenatal care is measured quantitatively, such as by the number of antenatal care visits attended or the completeness of case notes (e.g. the number of scans) [17]. Parents' perspectives of quality of care are not generally systematically captured and reported. Yet bereaved parents' experiences of sub‐optimal care is associated with stillbirth [15].

The inclusion of questions about antenatal care provision in our interview schedule, especially regarding information about quality of care and being heard, will enable, for the first time, parents' perceptions of maternity care to be systematically collected.

Questions proposed in the Fetal Wellbeing section included panellists' expert knowledge of warning signs that all might not be well with the fetus, including alterations in fetal behaviour [18], problems with placental supply and cord characteristics [19], and change in growth trajectory [20]. They also supported previous reports that stillbirth risk is reduced when maternity care providers give information about stillbirth risk during routine antenatal care provision [21].

Unsurprisingly, in Round 2, our panellists firmly endorsed parents being asked about fetal wellbeing. This endorsement lends weight to reports suggesting care providers may be unnecessarily concerned that providing information about reducing stillbirth risk during pregnancy may make women anxious [22]. However, panellists were careful to emphasise that questions such as these be asked with care to avoid attribution of blame while also gaining vital information regarding COD, as evidenced in Table 2 and the final schedule (see supplementary file).

In the maternal factors category, panellists again demonstrated their expert understanding of stillbirth risk factors by composing questions reflective of the current understanding of factors, such as the maternal going to sleep position [23], diet, activity and lifestyle choices such as avoiding certain foods and quitting smoking and alcohol while pregnant [24]. They also demonstrated understanding that the interplay between fetal vulnerability, maternal factors and fetal stressors are contributing factors to stillbirth [25]. For instance, a stressor could have no significant impact on a healthy fetus yet be a tipping point for a vulnerable fetus.

Furthermore, maternal factors such as hypertension, gestational diabetes mellitus, pre‐eclampsia and anaemia can each increase the risks of stillbirth [21]. While these diagnoses could be captured through clinical examinations both before and after pregnancy has ended, such as ultrasound assessments and biochemical tests, they may not be reported to the care provider during the pregnancy if the pregnant person did not recognise the significance of the symptoms [26].

Several panellists also highlighted their awareness of research regarding maternal intuition [27], that we should include a question about a “gut feeling” that something was wrong during the pregnancy, as shown in Table 2. Once again, several panellists also expressed that “careful consideration” needs to be given to how these questions are worded in the interview so as not to imply blame.

Sixty‐seven responses from Round 1 and 90% positive consensus indicated the need to ask about changes in the days just prior to the stillbirth, including any perceived changes in fetal well‐being, any changes for the mother, and approaches they took when these changes were noticed. Previous reports support that perceived alterations in fetal behaviour [28] and maternal well‐being [29] may herald poor pregnancy outcomes such as stillbirth. Asking about these events in a standardised interview post stillbirth may highlight commonalities in parental experience and thereby enable identification of novel risk factors for future investigation.

Questions regarding COD were included in the proposed interview schedule. This section of the interview will include questions on what parents perceived may have caused the death, what they were told caused the death, the extent to which the parents' agreed or disagreed with what they were told caused the death, and anything parents perceived could have changed the outcome. This echoes a previous study reporting that parents may hold a different opinion about the COD than what they were told [28]. Furthermore, a systematic review of parents' recommendations on follow‐up appointments after stillbirth reported that parents wish to discuss the baby's COD with their care provider [24]. Conducting a standardised interview post‐stillbirth which includes COD questions may help satisfy this request.

A parent interview is seldom included in perinatal mortality reviews. Thus, parents' experiences and perceptions about their baby's death do not usually form part of the COD decision. However, parents often hold information that is not included in the clinical record and, therefore is not drawn upon to determine the COD. This interview schedule was co‐designed with a panel of international experts. Through the delivery and testing of this interview schedule planned in phase two of this study, we can explore symptoms mothers experienced and examine preventable risk factors that may not be documented in case notes. We could also determine the best time to conduct the interview. Administering a standardised interview schedule will provide opportunities to further explore the interplay between fetal and maternal wellbeing, antenatal care, and events during the last days prior to the stillbirth to provide a holistic understanding of COD and risk factors for future preventative measures.

### 4.1 Strengths and Limitations

The strength of this study is that most of the panellists were bereaved parents. Through a consensus driven approach parents voiced that they want health professionals to ask questions about their pregnancy and their unborn baby to help enhance the COD diagnosis. A limitation of this research is the demographics of the population. Due to the funder's social media presence in the USA, most panellists were bereaved parents from the United States. These findings may not therefore be generalisable to other countries. Additionally, in comparison to parents, there were fewer clinician and researcher panellists. Thus, there may be a lack of clinical expertise represented in the obtained data. Nevertheless, the questions the panellists provided are well supported by the current evidence for stillbirth risk and prevention strategies from research published across the globe.

## Conflicts of Interest

The authors declare no conflicts of interest.
